# Asymmetric Waveforms Decrease Lethal Thresholds in High Frequency Irreversible Electroporation Therapies

**DOI:** 10.1038/srep40747

**Published:** 2017-01-20

**Authors:** Michael B. Sano, Richard E. Fan, Lei Xing

**Affiliations:** 1 Stanford University Medical Center, Department of Radiation Oncology, Division of Radiation Physics, Stanford, CA, USA; 2 UNC / NCSU Joint Department of Biomedical Engineering, Chapel Hill, NC, USA.; 3 Stanford University Medical Center, Department of Urology, Stanford, CA, USA.

## Abstract

Irreversible electroporation (IRE) is a promising non-thermal treatment for inoperable tumors which uses short (50–100 μs) high voltage monopolar pulses to disrupt the membranes of cells within a well-defined volume. Challenges with IRE include complex treatment planning and the induction of intense muscle contractions. High frequency IRE (H-FIRE) uses bursts of ultrashort (0.25–5 μs) alternating polarity pulses to produce more predictable ablations and alleviate muscle contractions associated with IRE. However, H-FIRE generally ablates smaller volumes of tissue than IRE. This study shows that asymmetric H-FIRE waveforms can be used to create ablation volumes equivalent to standard IRE treatments. Lethal thresholds (LT) of 505 V/cm and 1316 V/cm were found for brain cancer cells when 100 μs IRE and 2 μs symmetric H-FIRE waveforms were used. In contrast, LT as low as 536 V/cm were found for 2 μs asymmetric H-FIRE waveforms. Reversible electroporation thresholds were 54% lower than LTs for symmetric waveforms and 33% lower for asymmetric waveforms indicating that waveform symmetry can be used to tune the relative sizes of reversible and irreversible ablation zones. Numerical simulations predicted that asymmetric H-FIRE waveforms are capable of producing ablation volumes which were 5.8–6.3x larger than symmetric H-FIRE waveforms indicating that *in vivo* investigation of asymmetric waveforms is warranted.

Irreversible electroporation (IRE) is an emerging cancer therapy which uses high intensity electrical pulses to focally ablate solid tumors^1^. Clinically, two or more needle electrodes are advanced around a target tumor. A series of approximately 100 electrical pulses, 1000 to 3000 V in amplitude and 50 to 100 μs in duration, are then delivered. These electrical pulses locally increase cell transmembrane potentials above a critical lethal threshold to create permanent nanoscale defects, which result in rapid cell death. IRE ablations exhibit a characteristic sub-millimeter transition between complete cell death to unaffected tissue due to the rapid change in electric field intensity near the electrodes.

Small cohort studies of IRE for the treatment of inoperable tumors smaller than 3 cm show complete response rates between 93 and 98%^2^,^3^. These favorable early clinical results have encouraged significant interest in the use of IRE for the treatment of liver^4^, pancreatic^5^,^6^,^7^, kidney^8^, prostate^9^,^10^,^11^,^12^,^13^, and brain^14^,^15^,^16^,^17^ tumors.

Though IRE is a promising emerging procedure, there are some clinical challenges which may impede widespread adoption of the therapy. The long duration electrical pulses create local and systemic muscle contractions and may inadvertently interact with cardiac rhythms^18^. To alleviate this, patients must receive significant doses of chemical paralytics and pulse delivery is synchronized with the heart-beat to ensure pulses are delivered during the absolute refractory period^19^. This is challenging since it significantly changes the clinical workflow in comparison to other focal therapies and can increase the overall treatment time. Additionally, the ablation zone produced by IRE is dependent on local dynamic electrical properties of the tissue and heterogeneities can potentially distort the electric field and produce irregular shaped ablations^20^.

High frequency irreversible electroporation (H-FIRE) is a new protocol designed to address these challenges. H-FIRE replaces the long duration monopolar IRE pulses (Fig. 1a) with a burst of alternating polarity pulses between 250 ns and 5 μs (Fig. 1b). The electrical conductivities of skin, dura, tendons, blood vessels, and glandular tissues appear to converge at frequencies around 1 MHz^21^ resulting in H-FIRE ablations which more closely match analytical predictions in simple tissue models^22^. Numerical analysis predict that these waveforms can short through skin and fat layers to produce more uniform ablations than IRE^23^. H-FIRE waveforms effectively eliminate muscle contractions in small animal models^24^ and these treatments have been used to treat spontaneous tumors in large animals without the need for general anesthesia or chemical paralysis^25^. H-FIRE treatments have been shown to significantly inhibit tumor growth in murine flank tumor models^25^ and it was recently demonstrated that H-FIRE treatments preferentially target malignant cells in co-cultured 3D tumor models^26^. Limitations of H-FIRE include the ablation size, which is typically smaller than with conventional IRE and the need for specialized electronics which can deliver the appropriate alternating polarity electrical pulses.

*In vivo,* IRE is typically observed in regions which are exposed to approximately 500–750 V/cm^14^,^27^,^28^. Preliminary studies indicate that for equivalent energy treatments the lethal electric field is higher for H-FIRE than for IRE treatments^25^,^26^,^29^. Using a 3D *in vitro* pancreatic tumor model, Arena *et al*. found a lethal threshold of 500 V/cm when 80 × 100 μs IRE pulses were delivered^30^. The lethal electric field thresholds for equivalent energy H-FIRE protocols in the same model were much higher, at 2022, 1687, 1070, and 755 V/cm for bursts containing 0.25, 0.5, 1, and 2 μs symmetric waveforms, respectively^25^. Given this increase in lethal threshold, to successfully treat 1–3 cm tumors, these H-FIRE protocols would require a pulse generator which outputs significantly higher voltages than the 3000 V maximum currently implemented in the clinic.

Given these practical limitations, we present an alternate approach to decrease lethal thresholds and increase ablation volumes through the use of asymmetric pulse waveforms. Three experiments were conducted to evaluate the implications of pulse asymmetry in electroporation therapies. First, to investigate the impacts of culture conditions on IRE and H-FIRE outcomes, U87 cells were grown in either 2D monolayer or 3D cultures and exposed to an equivalent delivered energy in several different waveforms. These preliminary experiments mirror clinical protocols which deliver 100 μs monopolar pulses 100 times. Second, to demonstrate clinical utility against multiple forms of cancer, the lethal thresholds for U87 and MDA-MB-231 BR3 cells were examined in response to a broad array of pulse waveforms, comparing duration of energized bursts and pulse asymmetry. Third, to investigate if waveform asymmetry has any relevant implications for electro-chemotherapy (ECT)^31^ or electro-gene therapy (EGT)^32^, reversible electroporation thresholds were evaluated by adding membrane impermeable propidium iodide (PI) to 2D monolayers of each cell type prior to treatment. In this third experiment, cell cultures were exposed to lower energy treatments with 100x bursts where each burst was energized for 50 μs. Finally, the reversible and lethal thresholds found *in vitro* were incorporated into a three-dimensional finite element model to predict the size and shape of ablations which would be created by H-FIRE waveforms if 3 kV pulses were delivered into live tissue through clinical electrodes. These models were then validated against ablations created in *ex vivo* liver tissue.

It was found that both 2D and 3D culture conditions adequately replicate IRE lethal thresholds found *in vivo* and that 2D cultures are acceptable for investigating H-FIRE protocols. Waveforms which incorporate asymmetry had lower lethal thresholds and result in larger ablation zones than symmetric H-FIRE waveforms (Fig. 1c–e). This indicates that the elevated lethal thresholds previously found in H-FIRE treatments are likely the result of waveform symmetry, and reduced lethal thresholds can be achieved with asymmetric H-FIRE waveforms. It was found that the differences between reversible electroporation and lethal thresholds were substantially larger for symmetric waveforms than for asymmetric waveforms indicating that ECT and EGT treatment protocols may benefit from the use of symmetric waveforms. Finally, numerical models using experimentally determined lethal thresholds indicate that asymmetric H-FIRE waveforms can be used to produce 5.8–6.3x larger ablation volumes than symmetric waveforms. These results indicate that waveform asymmetry is a useful new parameter which can be used to modulate treatment outcomes and *in vivo* investigation of these waveforms is warranted.

## Methods

### Pulse Delivery

**Figure 1:**
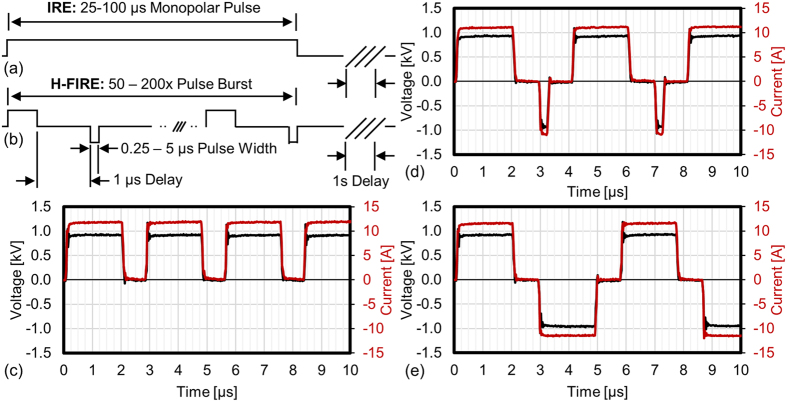
IRE and H-FIRE Use Different Pulse Waveforms: (**a**) IRE treatments deliver a series of long duration monopolar pulses. (**b**) H-FIRE treatments typically deliver multiple bursts containing 0.25 to 5 μs alternating polarity pulses. In this study we examine the effects of pulse symmetry. Example waveforms showing the first 10 μs of a (**c**) 2 μs monopolar, (**d**) asymmetric 2–1–0.25 μs (positive – delay – negative), and (**e**) symmetric 2–1–2 (positive – delay – negative) H-FIRE bursts. These waveforms are significantly more complex than standard IRE pulses and variations in the configuration of H-FIRE bursts impact treatment outcomes.

All treatments were delivered through two blunt 1.29 mm diameter (16 gauge) stainless steel needles with a 3 mm edge-to-edge separation. A custom pulse generator was used to deliver IRE and H-FIRE protocols which were either monopolar: all pulses were positive polarity (Fig. 1c), asymmetric bipolar: positive and negative pulses had different durations (Fig. 1d), or symmetric bipolar: positive and negative pulses were the same duration (Fig. 1e). To simplify discussion of these protocols we use the notation P - 1 - N where P indicates the positive pulse length and N indicates the negative pulse length in microseconds. All protocols used a 1 μs delay between changes in pulse polarity.

For example, 2–1–0.25 represents a pulse waveform with a 2 μs positive pulse, followed by a 1 μs delay, then a 0.25 μs negative pulse as shown in Fig. 1d. To compare equivalent energy IRE and H-FIRE treatments, the H-FIRE waveforms were repeated in a burst such that the total energized time of each burst was equivalent to 50 or 100 μs. In cases where waveforms could not produce energized times of exactly 50 or 100 μs, the closest energized time without exceeding the target time was used. In all treatment groups bursts were delivered with a repetition rate of 1 Hz and the output of the generator was set to deliver pulses which were 900 V in amplitude.

### Cell Culture

*In vitro e*xperiments were conducted on a glioblastoma cell line (U87, American Type Culture Collection Inc. Manassas, VA) and cells isolated from murine brain metastases after inoculation with human breast cancer cells (MDA-MB-231 BR3, a generous gift from the Laboratory of Martin Brown, Stanford, CA)^33^. These cells were chosen to model primary and metastatic brain tumors, respectively, and to evaluate potential changes in treatment response by cells of different disease origins. The cells were cultured to 80% confluency in High Glucose Dulbecco’s Modified Eagle Medium with L-Glutamine (catalog number 12800-082, Invitrogen Inc., Carlsbad, CA), which was supplemented with 10% heat inactivated Fetal Calf Serum (catalog number S11150, lot G14092, Atlanta Biologicals Inc., Lawrenceville, GA), 0.2 g/L Streptomycin Sulfate (catalog number 11860-038, Invitrogen Inc., Carlsbad, CA), and 0.126 g/L Penicillin G Potassium Salt (catalog number P7794-100MU, Sigma-Aldrich Inc., St. Louis, MO). Cells were harvested via trypsinization and seeded either directly onto the bottom of 12 well plates (2D culture) or in a 50% mixture of Matrigel (catalog number 356235, Corning Inc., Corning, NY) and culture media (3D culture) at a density of 100 k cells/mL. These cells were then allowed to reach confluent cultures over 4–7 days over which time cell culture media was replaced as necessary. Prior to treatment, media was removed and replaced with 2 mL of fresh culture media.

### Assessment of Reversible and Irreversible Electroporation

**Figure 2:**
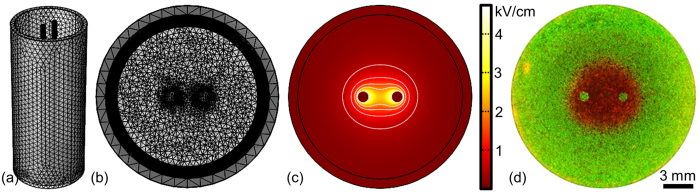
Pulsed Electric Fields Affect Cells within a Well Defined Treatment Zone which is Dependent on the Electrode Configuration and Pulse Parameters. (**a,b**) Mesh used to simulate the electric field distribution within a 12-well when 1 mm diameter electrodes spaced 3 mm edge-to-edge are used to deliver 900 V. (**c**) Electric field distribution within the well. Isocontour lines represent electric fields of 500, 1000, 1500, and 2000 V/cm [outside to inside]. (**d**) Electroporated [Red] and unaffected [Green] MDA-MB-231 BR3 cells immediately post-treatment after exposure to 100x H-FIRE Bursts. Each burst used a 2–1-0.5 μs waveform (positive – delay – negative time) which was energized for 50 μs. These numerical simulations were used to determine the electric field required to produce the focal treatment zone created *in vitro*.

**Figure 3:**
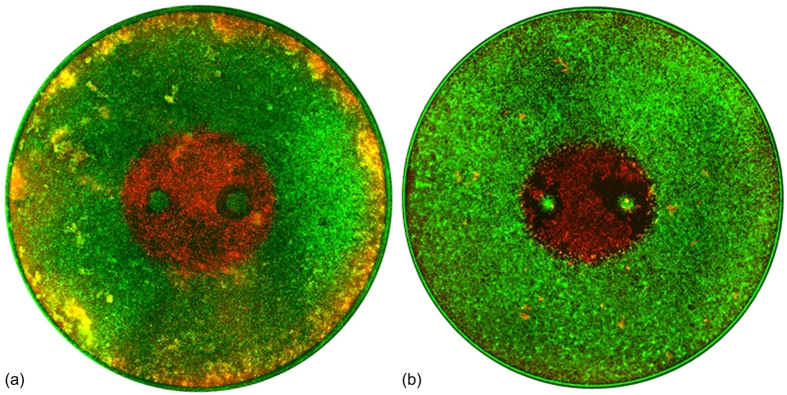
Comparison of ablations in 3D and 2D culture conditions. U87 cells were grown in (**a**) 3D Matrigel or (**a**) 2D media-only culture conditions. After reaching confluency cells were exposed to H-FIRE protocols. Live (green) and dead (red) images were obtained 24 hours post treatment to determine the ablation geometry. For most treatment groups, there was no statistical difference in the lethal threshold between 2D and 3D culture. Regions of live and dead cells are visible around the perimeter of the 3D cultures where the Matrigel forms a thick meniscus which may result in hypoxic conditions^30^. This comparative study indicates that 3D culture conditions may not be necessary to evaluate the relative effects of electroporation parameters *in vitro*.

Stock solutions of 4 μM Calcein AM (Life Technologies, Carlsbad, CA) were prepared by adding 125 μL of sterile dimethyl sulfoxide to 50 μg of powdered dye and stored at −20 °C until use. Stock solutions of propidium iodide (PI) (MP Biomedicals Inc., Burlingame, CA) were prepared using a concentration of 1 mg/mL PI in sterile phosphate-buffered saline and stored at 4 °C until use. Reversible electroporation thresholds were assessed by adding 100 μL/mL of PI stock solutions to each well 5 minutes prior to treatment. The samples were imaged immediately after treatment using a fluorescence microscope with an automated stage (Leica DMI600 B, Buffalo Grove, IL) using a red fluorescent protein filter cube (540–552 nm/580–620 nm) to visualize the electroporated population which had been counter-stained red by the PI (Fig. 2d). The height (y-axis) and width (x-axis) of each treatment zone was measured using the microscope software’s built in measurement tool. To assess lethal thresholds, cells were treated with IRE or H-FIRE protocols and incubated at 37 °C in 5% CO_2_ for 24 hours prior to staining. Calcein AM (2 μL/mL) and PI (100 μL/mL) stock solutions were then added to the cells and the ablation zone was imaged using green fluorescent protein (460–500 nm/512–542 nm) and red fluorescent protein filter cubes (Fig. 3) and measured using the microscope software’s built in measurement tool.

### Numerical Calculation of Reversible and lethal thresholds

To calculate the reversible electroporation or lethal threshold for each experimental treatment, the electric field distribution within a standard 12 well chamber was modeled numerically using COMSOL Multiphysics (V5.2a, COMSOL Inc., Palo Alto, CA) via the 3D Electric Currents module. This module solves the equations: 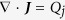, 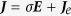, and 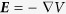 where **J** is the local current density, Q is the electric charge, **E** is the electric field, **J**_**e**_is the external current density, and V is the local voltage. A free tetrahedral mesh was generated in the media domain using the *Extremely Fine* preset while all other domains were meshed using the *Normal* preset values. The media domain was then refined twice using a refinement method which splits each tetrahedron along the longest edge (Fig. 2a,b). Experimental voltages were applied to the top most surface of one electrode. The top surface of the adjacent electrode was set to ground (0 V). All other external boundaries were set as insulators (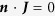). The electrical conductivity (σ) was set to 1.2 S/m for the media, 2.22 × 10^6^ S/m for the electrodes, and 1 × 10^−16^ S/m for the plastic well plate components. These simulations required approximately 29 minutes to solve on an Intel i7 processor with 32 GB RAM.

The electric field distributions (Fig. 2c) along the x- and y-axis were exported into a spreadsheet where they were used to correlate treatment geometries (Fig. 2d) with electric field values. Each treatment zone was measured and an electric field value correlating the height and width were determined. Each experimental parameter was repeated a minimum of three times (N = 3) yielding at least six electric field values which were averaged and reported as mean ± standard deviation. Statistical significance between groups was determined using a two-sided Student’s T-test with unequal variances. An alpha value of 0.01 (α = 0.01), 99% confidence interval, was used to determine if thresholds between treatment groups were statistically significantly different.

Finite element models were used to predict clinical ablations created by different H-FIRE waveforms (Supplemental Figure S1). These models used a dynamic tissue conductivity function^8^,^14^,^34^ with tissue conductivities derived from *in vivo* experiments^11^ and transitions between these conductivities centered at the reversible electroporation thresholds for each waveform found in this study. Methods for producing these models are presented in the supplemental material.

To validate these numerical models, predicted ablations were compared to ablations created in *ex vivo* liver tissue using NanoKnife electrodes (AngioDynamics Inc., Latham, NY). To match the simulations, a single needle and grounding pad configuration^35^ was used. *Ex vivo* porcine livers were obtained from the Stanford University Veterinary Service Center Necropsy Laboratory in accordance with the University’s guidelines and regulations. Whole organs were submerged in a water tank containing a 3 g/L NaCl solution with a conductivity of 0.6 S/m. A single electrode was inserted into the tissue and a 0.254 mm aluminum sheet was placed 10 cm from the liver to serve as a current sink, simulating a distant grounding pad. 100 × 2-1-2 H-FIRE bursts with each burst energized for 100 μs at approximately 3.5 kV were delivered to a minimum of three locations for each electrode configuration tested. The experiments were repeated using two different clinical probes for the NanoKnife, the “monopolar probe” (MP probe), a 1 mm diameter 1 cm long electrode and the “bipolar probe” (BP probe) which contains two 0.7 cm electrodes on a single 1.27 mm diameter probe. For the BP probe, the distal electrode was energized and the proximal electrode was disconnected. All experiments were conducted within four hours post-mortem. The tissue was sectioned and soaked in a 10 g/L tetrazolium chloride (TTC) solution for 20 minutes to dye metabolically active regions red and expose regions of tissue which had been irreversibly electroporated (Supplemental Figure S2). The length and width of the ablation zones were then measured using calipers and values were reported as mean ± standard deviation (Supplemental Figure S3).

## Results

### 2D versus 3D Culture Conditions

**Figure 4:**
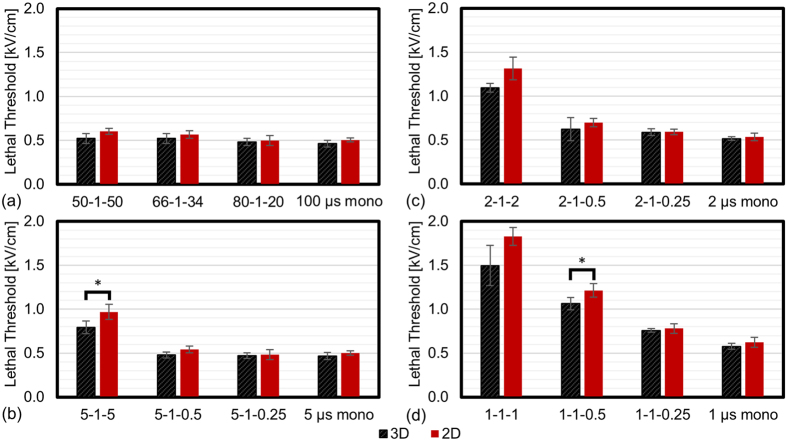
2D vs 3D Culture Conditions Had Minimal Impact on Lethal Threshold for U87 cells. Examination of lethal threshold values for cells cultured in 2D and 3D environments when exposed to 100x bursts energized for 100 μs. (**a**) Lethal threshold for long duration (>20 μs) IRE pulses which were either monopolar (100 μs) or alternated with a 1 μs delay between polarity change. Lethal thresholds for monopolar, symmetric, and asymmetric H-FIRE pulses where the longest pulse was (**b**) 5 μs, (**c**) 2 μs, and (**d**) 1 μs. For H-FIRE treatments, symmetric waveforms had significantly higher lethal threshold than the asymmetric and monopolar waveforms. This trend existed for both 2D and 3D cultures. *Indicate groups which are statistically significantly different (α = 0.01). Error bars represent one standard deviation from the mean. These results indicate that 3D culture conditions may not be nessary when evaluating the relative effects of electroporation pulse parameters *in vitro*.

A total of 16 protocols were examined in both 2D monolayer cultures and 3D Matrigel cultures (Fig. 4). All treatments delivered 100x bursts with each burst energized for a total of 100 μs. In 14 of 16 groups there was not a statistically significant difference (α = 0.01) between the lethal thresholds found in 2D and 3D culture platforms. In the two treatments which were statistically different, the mean lethal thresholds differed by an average of 18%. In groups which were not found to be statistically different, the mean lethal thresholds differed by an average of 9%. For U87 cells exposed to 100 μs monopolar IRE pulses, the lethal thresholds were 464 ± 38 V/cm and 505 ± 23 V/cm for 3D and 2D culture, respectively. Ivey *et al*. found a lethal threshold of 492 ± 41 V/cm for U87 cells treated with an identical IRE protocol when cells were cultured in 3D gels made with a 2% collagen mixture^26^ rather than the Matrigel material used in this study. These results indicated that culture conditions had a relatively small impact on lethal thresholds in comparison to other experimental parameters (e.g. waveform symmetry). 2D culture protocols were substantially less complex and less expensive than those required for producing and maintaining 3D cultures. Based on these factors, 2D culture conditions were used for all additional experiments to maximize the number of experimental parameters which could be evaluated.

### Effect of Waveform Asymmetry on Lethal Thresholds

The lethal thresholds for all waveforms and cell types evaluated in 2D culture are presented in Table 1. Introducing asymmetry into the pulse waveform produced measurable decreases in lethal thresholds. This effect was the largest for waveforms in which the longest pulse was 1 μs in duration (e.g. 1-1-1 vs. 1 μs mono) and became less prominent as pulses became longer in duration.

For U87 cells in 2D culture, the symmetric 50-1-50 and monopolar 100 μs waveforms had lethal thresholds of 603 ± 34 V/cm and 505 ± 23 V/cm, respectively (Fig. 4a). A relative decrease of 98 V/cm. In contrast, the symmetric 5-1-5 and the monopolar 5 μs waveform H-FIRE waveforms had lethal thresholds of 967 ± 88 V/cm and 499 ± 26 V/cm, respectively. A relative decrease of 468 V/cm. Similar results were found for the 2 μs (Fig. 4c) and 1 μs (Fig. 4d) protocols. It is interesting to note that the monopolar 5 μs and 2 μs waveforms had lethal thresholds which were not significantly different (α = 0.01) than the monopolar 100 μs waveform while the 5 μs and 2 μs symmetric waveforms had substantially higher lethal thresholds than the monopolar 100 μs waveform.

When the longest pulse in the waveform was either 1 μs or 2 μs, introducing asymmetry into the waveform by using a 0.5 μs pulse decreased the lethal threshold by an average of 25% and using a 0.25 μs pulse decreased the lethal threshold by an average of 45%, compared to the associated symmetric (1-1-1 or 2-1-2) waveforms. Use of a monopolar waveform decreased the lethal threshold by an average of 56% compared to the associated symmetric waveforms. All asymmetric H-FIRE treatment groups which were monopolar or incorporated 0.25 μs pulses had statistically significantly lower (α = 0.01) lethal thresholds than the associated symmetric waveforms. Additionally, the lethal thresholds for U87 cells exposed to 1-1-0.5 and 2-1-0.5 waveforms were significantly lower than those exposed to 1-1-1 and 2-1-2 waveforms, respectively.

### 50 μs versus 100 μs Energized Times

**Figure 5:**
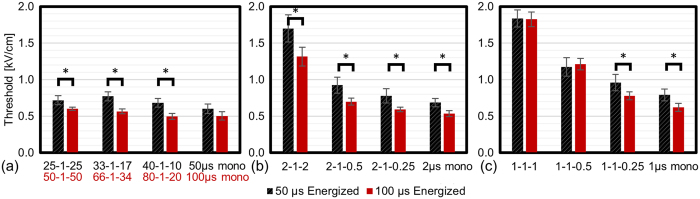
U87 lethal thresholds for treatments with 50 μs and 100 μs bursts. (**a**) Lethal threshold for long duration IRE pulses which were either monopolar (50 μs or 100 μs) or alternating polarity with a 1 μs delay between polarity change. Lethal thresholds for monopolar, symmetric, and asymmetric H-FIRE pulses where the longest pulse was (**b**) 2 μs and (**c**) 1 μs. All treatment groups received 100x bursts where each burst was energized for 50 μs (black) or 100 μs (red). *Indicate groups which are statistically significantly different (α = 0.01). Error bars represent one standard deviation from the mean. While doubling the energy delivered significantly decreased the lethal threshold for a majority treatment groups, waveform symmetry had a larger effect.

IRE is often employed clinically when tumors are in close proximity to critical nerves or blood vessels where tissue heating can result in deleterious outcomes. A simple way to minimize heating is to reduce the pulse-length (IRE) or energized time per burst (H-FIRE). Figure 5 shows the impact that reducing the energized time from 100 μs to 50 μs has on lethal thresholds for the U87 cell lines. The results for both cell lines are summarized in Table 1. The IRE waveforms in Fig. 5a which were energized for 100 μs had lethal thresholds which were 20% lower on average than those energized for 50 μs.

For H-FIRE treatments (Table 1), most treatment groups which received bursts energized for 100 μs had statistically significantly lower lethal thresholds than the 50 μs groups. However, this increase in energized time did not impact the lethal thresholds for U87 cells treated with 1-1-1 and 1-1-0.5 waveforms. Despite the relatively small differences found for these two waveforms, the lethal thresholds found for 100 μs H-FIRE treatments were 20% lower than those energized for 50 μs when averaged across all H-FIRE waveforms.

### Reversible Electroporation Thresholds

**Figure 6:**
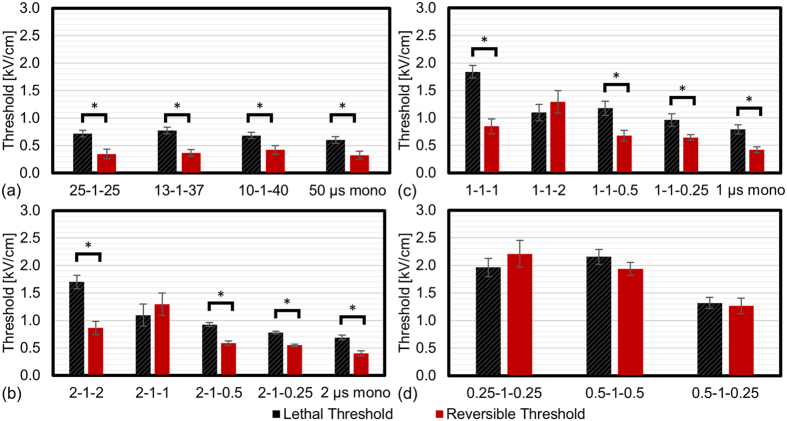
Pulse waveforms can be used to tune reversible and irreversible electroporation responses. Examination of reversible (red) and lethal (black) thresholds in 2D monolayer cultures of U87 cells for different 50 μs protocols. (**a**) Reversible and lethal thresholds for long duration (>20 μs) IRE pulses which were either monopolar (50 μs) or alternated with a 1 μs delay between polarity change. Reversible and lethal thresholds for monopolar, symmetric, and asymmetric H-FIRE pulses where the longest pulse was (**b**) 2 μs, (**c**) 1 μs, and (**d**) 0.5 μs. *Indicate groups which are statistically significantly different (α = 0.01). Error bars represent one standard deviation from the mean. When the therapeutic intent is to induce reversible electroporation while minimizing cell death, 1-1-1 and 2-1-2 symmetric H-FIRE waveforms may be favorable due to the large difference between the reversible and irreversible electroporaiton thresholds for these waveforms.

The reversible electroporation thresholds observed immediately after treatment are presented in Table 2 for both cell types and are shown in Fig. 6 for U87 cells. For the 50 μs monopolar, 25-1-25, 13-1-37, and 10-1-40 waveforms (Fig. 6a), the average reversible electroporation threshold of 303 V/cm was 40% lower than the lethal threshold when averaged across both cell types. There was not a statistically significant difference between the reversible electroporation thresholds between these waveforms.

Interestingly, there was some variation in reversible electroporation thresholds with H-FIRE waveforms (Fig. 6b–d). The largest differences between reversible and lethal thresholds occurred for the 1-1-1 and 2-1-2 symmetric bipolar waveforms. The 2-1-2 waveform resulted in the largest difference of 837 V/cm for U87 (49% lower). In contrast, the 2 μs monopolar waveform only had a difference of 289 V/cm between the lethal and reversible electroporation thresholds and there was not a statistically significant difference for the 1-1-2 waveform for either cell type.

For 1 μs H-FIRE waveforms (Fig. 6c), the 1-1-1 waveform resulted in the largest difference of 992 V/cm (54% lower) for U87 cells between the reversible and lethal thresholds. The smallest difference between these two thresholds of 315 V/cm was found for the 1-1-0.25 waveform.

Sub-microsecond H-FIRE protocols were also evaluated (Fig. 6d). For U87 cells there was no statistically significant difference between the reversible and lethal thresholds for the 0.25-1-0.25, 0.5-1-0.5, and 0.5-1-0.25 waveforms. However, the asymmetric 0.5-1-0.25 waveform had lower reversible and irreversible thresholds than the 0.25-1-0.25 and 0.5-1-0.5 symmetric waveforms.

### U87 vs MDA-MB-231 BR3 Cell Lines

The lethal thresholds for U87 cells were lower than for MDA-MB-231 BR3 cells for all waveforms in Table 1, the mean thresholds were 36% lower for U87 cells. The largest difference observed (137%) was for the 1-1-0.5 waveform when 100 × 50 μs bursts were delivered. The smallest difference (13%) was observed for the 33-1-17 waveform. The 50 and 100 μs IRE groups in Fig. 5a had the smallest average difference in lethal threshold between cell types at 26%. The average difference between cell types increased to 31% for 2 μs (Fig. 5b) and 49% for 1 μs (Fig. 5c) H-FIRE waveforms.

The reversible electroporation thresholds were lower for U87 cells than for MDA-MB-231 BR3 cells in 11 of 16 waveforms evaluated in Table 2. Only the 0.5-1-0.5 waveform had a reversible electroporation threshold for U87 cells which was statistically significantly greater than for MDA-MB-231 BR3 cells. The 2-1-2, 1-1-2, 0.5-1-0.25, and 0.25-1-0.25 waveforms had also had mean reversible electroporation thresholds which were higher for the U87 cells, however these were not statistically significant different between cell types.

### Modeling the Impact of Pulse Asymmetry on Clinical Ablations

Finite element models which incorporate the reversible and lethal electric field thresholds found here were used to predict how different H-FIRE waveforms may affect the size and geometry of ablations created using clinical ablation electrodes and pulse voltages used in the treatment of solid tumors. The methods for creating these models and validation against ablations created in *ex vivo* liver tissue are presented in the supplemental document. Symmetric H-FIRE waveforms required significantly higher electric fields to induce cell death compared to asymmetric waveforms. This resulted in the production of smaller predicted ablations for symmetric waveforms compared to the asymmetric waveforms when calculated using experimentally determined lethal thresholds (Supplemental Figure S1c-g).

A simulated treatment delivering 100 × 3.0 kV 50 μs monopolar pulses through a 1 cm electrode exposure (MP Probe) predict an ablation measuring 1.7 × 1.0 cm. For the same 50 μs energized time, the 2 μs monopolar waveform produced the largest simulated H-FIRE ablation of 1.6 × 0.9 cm followed by 1.6 × 0.8 cm, 1.5 × 0.7 cm, and 1.3 × 0.4 cm for the 2-1-0.25, 2-1-0.5, and 2-1-2 waveforms, respectively (Supplemental Table S1). Volumetrically, ablations created by the 2 μs monopolar waveforms were 6.3x larger than the symmetric 2-1-2 waveform.

Increasing the energized time from 50 μs to 100 μs resulted in a decrease in lethal threshold of approximately 20% averaged across all waveforms evaluated in this study. 1.8 × 1.1 cm, 1.7 × 1.0 cm, 1.7 × 1.0 cm, 1.6 × 0.9 cm, 1.4 × 0.5 cm simulated ablation zones were created for the 100 μs mono (505 V/cm), 2 μs mono (536 V/cm), 2-1-0.25 (594 V/cm), 2-1-0.5 (700 V/cm), and 2-1-2 (1316 V/cm) waveforms, respectively. Under these conditions, the 2 μs monopolar waveform produced a simulated ablation volume which was 5.8x larger than the symmetric 2-1-2 waveform.

### *Ex Vivo* Results versus Numerical Predictions

Experimental ablations were created in *ex vivo* porcine liver by delivering 100 × 3.5 kV bursts of the 2-1-2 waveform (Supplemental Figure S2). Each burst was energized for 100 μs and bursts were delivered at a repetition frequency of 1 Hz. Experimental ablations created using the clinical MP probe with a 1 cm electrode exposure had an average length of 1.3 ± 0.3 cm and an average width of 0.7 ± 0.2 cm (Supplemental Figure S3). The numerical model of the MP probe predicted an ablation measuring 1.4 × 0.6 cm when these experimental conditions were simulated (Supplemental Table S2) and the results were within 0.1 cm of the average ablation created experimentally (1.3 × 0.7 cm). Experimental ablations created using the clinical BP probe had an average length of 1.4 ± 0.1 cm and an average width of 0.9 ± 0.2 cm (Supplemental Figure S3). The numerical model of the BP probe predicted an ablation measuring 1.2 × 0.7 cm using these experimental parameters (Supplemental Table S3) and the results were within 0.2 cm of the average ablation created experimentally (1.4 × 0.9 cm). This difference of one to two millimeters in length and width between the average experimental and predicted ablations serve as a preliminary validation of the ability of these models, which incorporate dynamic tissue conductivity with reversible and lethal thresholds found *in vitro*, to predict ablation dimensions in tissue.

## Discussion

### 2D versus 3D Culture Conditions

H-FIRE is a relatively new electroporation protocol. The symmetric waveforms were first proposed by Arena *et al*. as a method for creating more uniform ablations in heterogeneous tissues^23^. Early *in vitro* experiments on cells in suspension showed that these waveforms required substantially higher electric fields (2–2.7x) to induce cell death versus the standard 100 μs monopolar IRE waveforms^29^. When cells were grown in 3D collagen tumor models the lethal electric field threshold decreased to 1.5x and 2.2x the lethal threshold for equivalent energy 2 μs and 1 μs symmetric H-FIRE protocols, respectively. It is hypothesized that the decrease in the ratios of lethal thresholds between IRE and H-FIRE protocols is likely due to cells transforming from a spherical shape in suspension into a more complex natural geometry when cultured in 3D models^29^.

Arena *et al*. found a lethal threshold of 500 V/cm for pancreatic cancer cells when delivering 80 × 100 μs monopolar IRE pulses in this 3D model^30^. The lethal thresholds for U87 brain cancer cells found in this study were 463 V/cm and 505 V/cm in 3D and 2D models, respectively, when 100 × 100 μs monopolar IRE pulses were delivered. Previous studies found lethal thresholds in liver tissue between 300 and 640 V/cm using similar IRE protocols^36^,^37^,^38^, indicating that both the 2D and 3D culture models recapitulate effects seen in mammalian tissue. Fourteen of sixteen protocols conducted in 2D and 3D conditions showed no statistically significant difference in lethal threshold indicating that the 3D model has a limited effect on the lethal threshold and cells cultured in a 2D monolayer are a relatively effective model for studying H-FIRE. 2D monolayers are a convenient model as they are relatively simple to generate, are less expensive, and less time intensive to create compared to 3D cultures. However, when the thresholds were statistically different between groups, the 3D cultures all had lower lethal thresholds. This indicates that further investigation in 3D constructs may be warranted, especially if *in vivo* results are found to differ significantly from those found in 2D monolayers.

### U87 vs MDA-MB-231 BR3 Cell Lines

The MDA-MB-231 BR3 cells appeared to stretch out into a flattened oval shape with few protrusions. These cells continued to divide as the cultures approached confluency and appeared to overlap with each other to cover the entire well plate surface. In contrast, the U87 cells formed more star shaped geometries with multiple elongated protrusions. They formed a web of interconnected cells and cell division slowed as they became confluent. The networks of U87 cells had numerous gaps of unoccupied well plate area. While not extensively examined here, the differences in lethal thresholds observed between cell types may be due to differences in cell geometry affecting the charging time of the cell membrane. Ivey *et al*. found identical lethal thresholds for U87, NHA, and D1TNC1 cell lines after treatment with 100 μs monopolar pulses, but significantly different lethal thresholds between U87 cells and the astrocyte (NHA and D1TNC1) cell lines when using a 1-5-1 H-FIRE waveform^26^. These differences in lethal threshold were hypothesized to be a function of the morphology of the cell including the nucleus-to-cytoplasm ratio^26^ and morphological factors may have impacted the observed differences between U87 and MDA-MB-231 BR3 cells shown here.

### Effects of Waveform Asymmetry and Energized Time

For H-FIRE, waveform asymmetry appeared to be the biggest factor affecting the lethal threshold. Doubling the dose delivered by energizing the bursts for 100 μs rather than 50 μs decreased the lethal threshold by an average of 20%. In contrast, the lethal thresholds for asymmetric waveforms were 42% lower than the symmetric waveforms, averaged across all H-FIRE groups. Waveforms incorporating a 0.5 μs or 0.25 μs pulses were 25% and 45% lower than symmetric waveforms, respectively. When only monopolar 1 μs or 2 μs pulses were used in the burst, the lethal thresholds were 56% lower on average compared to their respective symmetric (1-1-1 or 2-1-2) counterparts.

We hypothesize that two potential mechanisms contribute to the decrease in lethal threshold when asymmetric waveforms are used: a cumulative charging of the cell membrane and enhanced electrokinetic transport. Symmetric pulses may be inducing a cancellation effect^39^ in which the alternating polarity pulses sequentially charge then discharge the potential across the cell membrane faster than this potential would dissipate in the absence of an alternating pulse. The symmetric H-FIRE waveforms had the largest differences between reversible and lethal thresholds indicating that these waveforms are effective at facilitating pore formation, but the rapid charging and discharging of the membrane may not have efficiently driven the pore expansion processes necessary to permanently disrupt the cell membrane. When asymmetric waveforms are used, the forced discharge caused by the alternate polarity pulse occurs to a lesser extent than for symmetric waveforms resulting in a cumulative charging of the cell membrane by the longer duration pulses in the burst. This cumulative charging likely increases the potential across the cell membrane above the threshold required for pore formation and then then drives pore expansion.

Monopolar IRE pulses act as a baseline to indicate the lowest electroporation thresholds possible under these experimental conditions. Monopolar H-FIRE waveforms which contained 5 μs or 2 μs pulses had nearly identical lethal thresholds to equivalent 50 or 100 μs pulses. This indicates that the induced membrane potential does not decrease substantially during the 1 μs delay between subsequent pulses in a monopolar H-FIRE burst. However, 1 μs monopolar H-FIRE waveforms were less efficient at inducing cell death than 2 μs or 5 μs monopolar waveforms indicating that this may be the lower limit for inducing pore expansion.

This 1 μs threshold is likely related to the charging time of the cell membrane which is dependent on a number of factors including cell geometry and medium conductivity, but is typically calculated to be approximately 1 μs^29^,^40^. Previous studies on sub-microsecond pulses showed that increasing the number of monopolar pulses increased the number, but not the size of nanopores formed in the membrane^41^. A similar phenomena may be occurring here in which continued delivery of pulses 1 μs or shorter leads to the formation of numerous smaller pores rather than rapid cell membrane destruction caused by pore expansion and any lethal effects are due to mechanisms which occur post-treatment.

Symmetric pulses may also be inducing a cancelation effect which results in zero net movement of charged species. The introduction of asymmetry in the H-FIRE waveform may result in net electrokinetic transport of charged molecules across the cell membrane resulting in conditions which make it challenging for the cell to regain homeostasis. It was found that monopolar 300 ns pulses resulted in a higher degree of calcium mobilization and lower viabilities 24 hours post treatment compared to bipolar waveforms with inter-pulse delays of up to 10 μs^39^. Additionally, 100 μs monopolar pulses resulted in significantly higher magnitudes of PI influx compared to equivalent 1-1-1 H-FIRE waveforms^42^ for cells in suspension. A combination of these phenomena may be occurring with asymmetric H-FIRE waveforms resulting in the formation of larger pores due to pore expansion and increased molecular transport due to electrophoresis, however, the degree to which these phenomena occur have yet to be investigated for asymmetric H-FIRE waveforms.

### Reversible Electroporation

Typical ECT^32^ and EGT^43^,^44^ protocols use 2 to 24^45^ monopolar pulses between 100 μs^46^ and 20 ms^47^ to electroporate cells and electrophoretically drive molecules across the cell membrane. A challenge with these monopolar waveforms is that they induce some degree of irreversible electroporation *in vivo* due to the relatively small difference between their reversible and lethal thresholds. Previous studies by Miklavcic *et. al* found a difference of 275 V/cm between the reversible and lethal thresholds in *in vivo* rabbit liver tissue after the delivery of eight 10 μs pulses^27^ and the monopolar IRE pulses investigated in this study had approximately 225–277 V/cm between the reversible and lethal thresholds. From a clinical prospective, symmetric H-FIRE waveforms may be more advantageous in ECT and EGT protocols, where the intent is to transfer membrane impermeable material into the cell rather than inducing cell death, because the symmetric waveforms have a very large difference between their reversible and irreversible thresholds (837–1573 V/cm).

It is unclear why symmetric waveforms resulted in a larger difference between reversible and irreversible thresholds. However, we hypothesize that the rapid charging and discharging of the cell membrane by the symmetric pulses inhibits pore expansion and instead results in an increase in the number, but not size of the pores formed^41^. However, it is unclear if these symmetric waveforms will be capable of transporting molecules across biological membranes efficiently. Numerical simulations indicate that electrophoretic and diffusive transport of molecules play roughly equivalent roles in the transport of small molecules following electroporation^48^ while electrophoresis^49^ and electroporation induced endocytosis^50^ are implicated in the transport of larger molecules^51^.

Symmetric bipolar wave forms were recently reported to be an effective means for transiently disrupting the blood brain barrier^52^, however, reversible electroporation for chemotherapy and gene transfer have yet to be demonstrated *in vitro* or *in vivo* with these waveforms. We hypothesize that the introduction of a small degree of asymmetry into the H-FIRE waveform (e.g. a 2.0-1-2.1 waveform) may be sufficient to induce electrophoretic movement of charged molecules without producing large lethal ablation zones or inducing muscle contractions^53^. Andre *et al*. demonstrated that high voltage pulses to induce electroporation followed by low voltage pulses to enhance electrophoretic transport were advantageous for *in vivo* EGT^49^ and a similar strategy may be necessary with H-FIRE. However, further work will be necessary to determine optimal protocols for using H-FIRE waveforms for these non-lethal protocols.

### Modeling the Impact of Pulse Asymmetry on Clinical Ablations

The use of numerical models for predictive treatment planning is relatively common for IRE procedures^8^,^11^,^17^,^54^,^55^, in part because real time ultrasound visualization of the ablation zones is challenging and follow up CT or MRI imaging may be required to confirm total tumor coverage^56^. The model presented here builds upon previous dynamic conductivity models^11^,^17^,^34^ by incorporating reversible and lethal thresholds found *in vitro* into the dynamic conductivity function. Given recent data which indicates that H-FIRE may be able to preferentially target malignant cells^26^, this modeling approach may be necessary for predicting ablations margins created in *in vivo*.

Finite element models simulating clinical electrodes and treatment voltages (Supplemental Figure S1) indicate that asymmetric H-FIRE waveforms can potentially be used to produce equivalent ablation volumes to standard IRE waveforms (Supplemental Table S1). A 3 kV treatment using a 2-1-2 waveform energized for 100 μs produced a 1.4 × 0.5 cm simulated ablation (0.18 cm^3^ volume) as shown in Supplemental Figure S1g. In contrast, switching to a 2-1-0.25 waveform would increase the ablation volume 5.5-fold to 1.0 cm^3^ (1.7 × 1.1 cm).

Clinically, a single electrode and grounding pad configuration is not implemented because 100 μs pulses would stimulate muscles over such a wide of a volume of the body that chemical paralytics may no longer be sufficient to eliminate muscle contractions. Symmetric H-FIRE waveforms have been shown to mitigate these contractions^24^ and eliminate the need for chemical paralytics in large animal studies^25^ at the expense of creating smaller ablations^25^. Extensive *in vivo* investigation will be necessary to evaluate if muscle contractions are present when asymmetric H-FIRE waveforms are used or if chemical paralytics will be required to mitigate them. However, based on previous studies it is feasible that asymmetric H-FIRE waveforms will be able to produce clinically relevant ablations using a single electrode.

### Study Limitations

There are a number of important limitations to the current study. 2D and 3D *in vitro* cell cultures have been found to adequately recapitulate the lethal electric field thresholds required for IRE treatments to induce cell death *in vivo*. However, data on *in vivo* lethal thresholds for H-FIRE treatments have yet to be published and it is unknown if values determined *in vitro* will match those found *in vivo*. Here and in prior studies^25^,^30^, lethal thresholds for IRE and H-FIRE waveforms were investigated approximately 24 hours post treatment. The mechanisms of cell death following H-FIRE are unclear, but appear to be a combination of physical necrosis due to cell membrane destruction and apoptosis. However, mechanistic studies conducted for durations longer than 24 hours have yet to be reported. Preliminary studies on cells in suspension showed that viability after H-FIRE treatment continued to decline between 1 and 24 hours post treatment^29^ and it is unclear if the ablation zones created in 2D or 3D culture would continue to evolve over longer time periods. Theoretical clinical ablation zones were generated using numerical models which have previously been used for clinical treatment planning^17^ combined with experimental electroporation threshold data generated in 2D culture. These models predict that asymmetric waveforms may be useful in increasing the size of H-FIRE ablations *in vivo*, however, extensive experimental examination will be required to validate these results.

## Conclusion

*In vitro* models of primary and metastatic brain cancer were used to show that the lethal threshold for H-FIRE treatments is affected by the symmetry of the waveform. Asymmetric waveforms, including monopolar waveforms, have significantly lower lethal thresholds than equivalent energy symmetric waveforms. The use of asymmetric H-FIRE waveforms clinically should result in the creation of equivalent ablation volumes to those seen in IRE procedures while mitigating muscle contractions caused by long duration pulses. These results indicate that *in vivo* testing to determine anti-tumor efficacy of asymmetric H-FIRE waveforms is warranted.

## Additional Information

**How to cite this article:** Sano, M. B. *et al*. Asymmetric Waveforms Decrease Lethal Thresholds in High Frequency Irreversible Electroporation Therapies. *Sci. Rep.* **7**, 40747; doi: 10.1038/srep40747 (2017).

**Publisher's note:** Springer Nature remains neutral with regard to jurisdictional claims in published maps and institutional affiliations.

## Supplementary Material

Supplementary Information

## Figures and Tables

**Table 1:** Lethal thresholds for 2D confluent monolayers of U87 and MDA-MB-231 BR3 cells exposed to 100x bursts of the listed waveforms.

50 μs Energized			100 μs Energized		
Waveform	U87	MDA-MB-231 BR3	Waveform	U87	MDA-MB-231 BR3
	Threshold [V/cm]	Threshold [V/cm]		Threshold [V/cm]	Threshold [V/cm]
25-1-25	718 ± 61	879 ± 145	50-1-50	603 ± 34	725 ± 37
33-1-17	773 ± 63	876 ± 46	66-1-34	567 ± 42	693 ± 42
40-1-10	684 ± 60	906 ± 61	80-1-20	498^*^ ± 57	718 ± 20
50 μs Mono	603^*^ ± 63	779 ± 79	100 μs Mono	505^*^ ± 23	644 ± 55
2-1-2	1702 ± 186	2028 ± 63	5-1-5	967 ± 88	998 ± 55
2-1-0.5	925^*^ ± 112	1300^*^ ± 49	5-1-0.5	541^*^ ± 37	826^*^ ± 38
2-1-0.25	780^*^ ± 99	971^*^ ± 85	5-1-0.25	484^*^ ± 56	812^*^ ± 72
2 μs Mono	688^*^ ± 58	1005^*^ ± 89	5 μs Mono	499^*^ ± 26	732^*^ ± 82
1-1-1	1839 ± 116	2761 ± 209	2-1-2	1316 ± 129	1563 ± 221
1-1-0.5	1175^*^ ± 128	2782 ± 103	2-1-0.5	700^*^ ± 47	885 ± 57
1-1-0.25	960^*^ ± 114	1264^*^ ± 55	2-1-0.25	594^*^ ± 32	818^*^ ± 25
1 μs Mono	792^*^ ± 80	1051^*^ ± 82	2 μs Mono	536^*^ ± 43	732^*^ ± 46
			1-1-1	1827 ± 101	2271 ± 126
			1-1-0.5	1213^*^ ± 77	2000 ± 195
			1-1-0.25	780^*^ ± 55	945^*^ ± 59
			1 μs Mono	622^*^ ± 56	826^*^ ± 58

Each burst was energized for 50 or 100 μs and delivered at a repetition rate of 1 Hz. Values are listed as mean ± standard deviation. ^*^Indicates asymmetric waveforms which were found to be statistically significantly (α = 0.01) different from their respective symmetric waveforms.

**Table 2:** Reversible electroporation thresholds for U87 and MDA-MB-231 BR3 cell lines after exposure to 100x bursts of the listed waveforms.

Waveform	U87		MDA-MB-231 BR3	
	Threshold [V/cm]	Lethal Δ	Threshold [V/cm]	Lethal Δ
25-1-25	348 ± 87	370	569 ± 84	310
33-1-17	366 ± 61	407	606 ± 100	269
40-1-10	420 ± 78	264	605 ± 32	301
50 μs Mono	326 ± 70	277	554 ± 9	225
2-1-2	865 ± 122	837	864 ± 62	1164
2-1-0.5	587^*^ ± 36	338	679^*^ ± 34	621
2-1-0.25	548^*^ ± 23	232	593^*^ ± 57	378
2 μs Mono	400^*^ ± 45	289	476^*^ ± 50	528
1-1-1	848 ± 137	992	1188 ± 171	1573
1-1-0.5	679 ± 94	497	1330 ± 314	1452
1-1-0.25	645 ± 53	315	868 ± 208	395
1 μs Mono	422^*^ ± 53	371	579^*^ ± 41	472
0.25-1-0.25	2209^β^ ± 243	−248	2021^δ^ ± 84	967
0.5-1-0.5	1936^γ^ ± 117	218	1274 ± 59	1340
0.5-1-0.25	1263^βγ^ ± 143	55	1147^δ^ ± 226	520
1-1-2	1294 ± 202	−196	1102 ± 313	119

Each burst was energized for 50 μs and delivered at a repetition rate of 1 Hz. Lethal Δ indicates the difference between the irreversible and reversible thresholds. Values are listed as mean ± standard deviation. ^*^Indicates asymmetric waveforms which were found to be statistically significantly (α = 0.01) different from their respective symmetric waveforms. ^β^, ^γ^, and ^δ^ symbols indicate sub-microsecond groups which were found to be statistically different from other sub-microsecond groups marked with the same symbol.
