# Supplementary material for: Asymmetric Waveforms Decrease Lethal Thresholds in High Frequency Irreversible Electroporation Therapies
Source: Sci Rep. 2017 Jan 20;7:40747. doi: 10.1038/srep40747 (PMC5247773; doi:10.1038/srep40747)
Supplement: Supplementary Information [file srep40747-s1.pdf]

Asymmetric Waveforms Decrease Lethal Thresholds in High Frequency Irreversible Electroporation Therapies

Michael B. Sano[1,*], Richard E. Fan[2], Lei Xing[1]
[1]Stanford University Medical Center, Department of Radiation Oncology, Division of Radiation Physics
[2] Stanford University Medical Center, Department of Urology

Modeling the Impact of Pulse Asymmetry on Ablations
An irreversible electroporation threshold of 637 ± 43 V/cm was established by Miklavcic et al.[1] who compared the isocountour lines from finite element simulations to histological findings in rat liver tissue which had been exposed to eight 100 µs monopolar electrical pulses of various voltages. Davalos et al. then demonstrated, via finite element simulations, the feasibility of using irreversible electroporation as a minimally invasive, non-thermal ablative procedure[2]. These models used a static electrical conductivity, one which does not change as a result of reversible electroporation or thermal changes in tissue, and an irreversible electroporation threshold of 680 V/cm to predict the volume of tissue which would undergo no-thermal ablation under a variety of voltages and needle electrode configurations[2]. Subsequent *in vivo* studies on canine kidneys by Neal et al. and canine brain by Garcia et al. demonstrated that the use of a sigmoidal dynamic conductivity function, developed by Sel et al.[3],which increased from a baseline conductivity to an 'electroporated conductivity' produced electric field isocountours (kidney: 575 ± 67 V/cm [4], brain: 502 ± 8 V/cm [5]) which most accurately matched *in vivo* lesion dimensions. Rossmeisl et al. used this dynamic conductivity finite element modeling method to successfully conduct preoperative treatment planning on seven canine patients with spontaneous gliomas[6]. These results build upon the work of Miklavcic et al.[7-10] and highlight the utility of this dynamic conductivity modeling technique and its ability to both derive data relating to electroporation thresholds and accurately predict ablation zones for new electrode configurations and treatment protocols.

Here we use a dynamic conductivity finite element model, which incorporates the reversible and irreversible thresholds found *in vitro* with dynamic conductivity data found *in vivo*[11] to demonstrate the potential impact of various H-FIRE waveforms on clinical ablation sizes using a single electrode and grounding pad configuration.

Electrically induced muscle contractions are an unintended consequence during IRE and may result in complications if the electrodes move during treatment. Organ translocations of 3 to 5 cm have been reported in

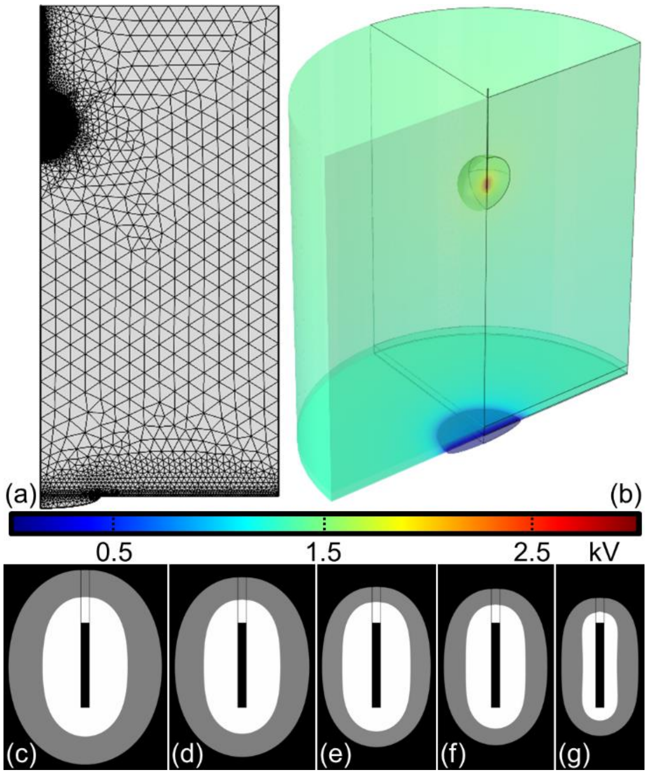

Supplemental Figure S1: Impact of asymmetric H-FIRE protocols on in vivo irreversible and reversible electroporation protocols. (A) Finite element mesh used to calculate the electric field distribution within a simulated tissue domain using a single 1 cm long, 1 mm diameter needle electrode and distal grounding pad. (B) Voltage distribution during pulse delivery when 3kV is applied between the electrode and grounding pad. Comparison of lethal [white] and reversible [grey] ablation zones for (C) 50 µs mono, (D) 2 µs mono, (E) 2-1-0.25, (F) 2-1-0.5, and (G) 2-1-2 waveforms. Values for reversible and lethal thresholds were calculated using values determined experimentally from U87 cells exposed to 100x bursts of each waveform.

response to pulse delivery[12]. To prevent this, patients are anesthetized during IRE treatment using a strict protocol which includes the administration of a neuromuscular blockade which requires intubation and mechanical ventilation[12]. There is evidence that these neuromuscular blocks interfere with pharyngeal, pulmonary, and respiratory function[13] and in some cases mild local muscle contraction continues despite these preventative measures[14].

For 100 µs IRE pulses, the electric field required to induce muscle contractions is approximately 1.8 V/cm[15]. Muscle contractions during IRE are observed when the electrical source and sink are co-located within organs undergoing treatment and the surrounding muscle tissue is exposed to relatively low electric fields. For these long duration pulses, the use of a single source and a distal grounding pad may result in muscle contractions which cannot be inhibited using acceptable paralytic regimens, because a significantly larger volume of muscle tissue will be exposed to electric fields above the muscle contraction threshold.

The electric field required to induce muscle contractions increases dramatically for stimuli below 100 µs[15] and the use of bipolar waveforms additionally increases this threshold[16]. For 1 µs pulses, an electric field of approximately 112 V/cm is required to induce muscle contractions[17] and recent studies have shown that H-FIRE waveforms drastically reduce the intensity of muscle contractions *in vivo*[18] and enable the treatment of spontaneous tumors without neuromuscular blockades[19]. We hypothesize that the use of a single electrode within the treatment volume and distal grounding pad may be clinically possible with H-FIRE due to the increase in the electric field required to induce muscle contraction with these pulses.

Models of this single electrode and grounding pad configuration were constructed in COMSOL Multiphysics (V5.2a, COMSOL Inc., Palo Alto, CA). A two dimensional axisymmetric geometry (Supplemental Figure S1A) was used to simulate a three dimensional domain (Supplemental Figure S1B). A cylindrical geometry representing an abdominal cavity had a radius of 20 cm and height of 40 cm. A flattened spheroid geometry was used to simulate a grounding pad with a 5 cm radius contact region between the grounding pad and tissue. A 1 cm thick layer of fat ($\sigma = 0.027$ S/m) and a 1.5 mm thick layer of skin ($\sigma = 0.029$ S/m) separated the grounding pad from the tissue. 1 mm and 1.26 mm diameter cylindrical geometries were used to simulate the probe body ($\sigma = 1 \times 10^{-12}$ S/m) and exposed electrodes ($\sigma = 4.032 \times 10^{6}$ S/m) of two different clinically available electrodes for

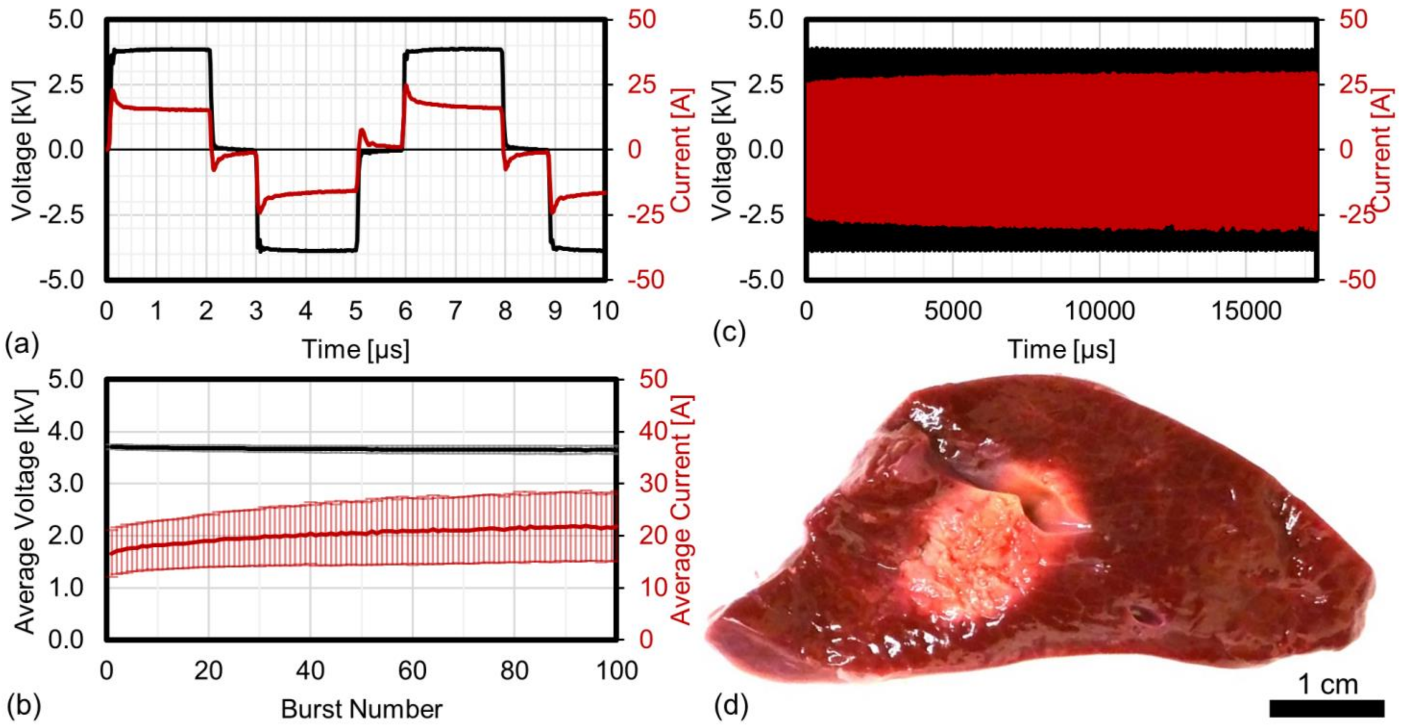

Supplemental Figure S2: Experimental ablations to validate numerical simulations. Ablations were created in *ex vivo* liver tissue using (A) 100x 3.5 kV 2-1-2 bursts where each burst was energized for 100 µs. (B) Voltage and current were averaged for each burst and the (C) entire treatment was recorded. (D) Ablations (white) were imaged by counterstaining the tissue with a metabolic dye (red).

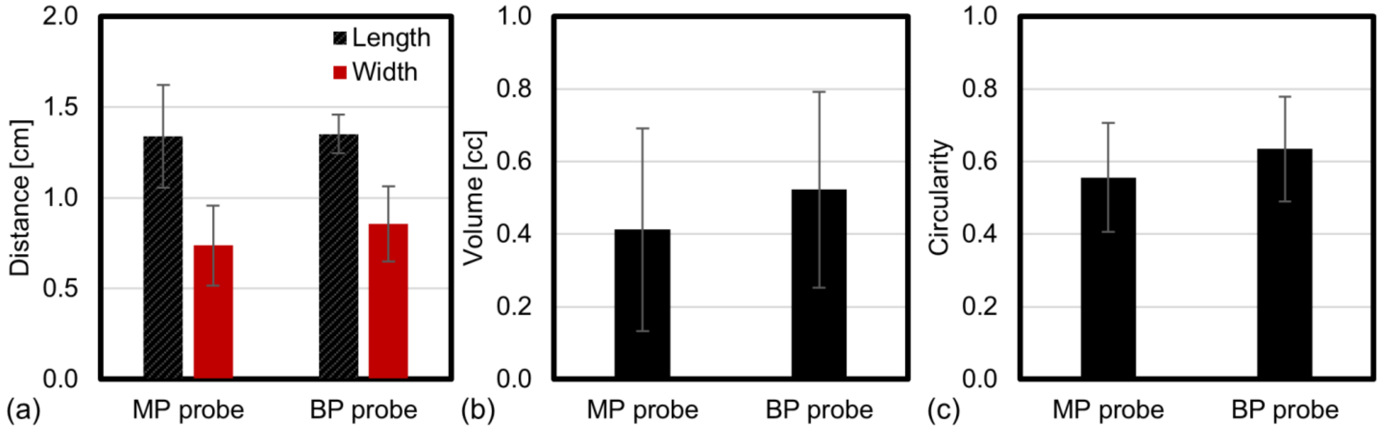

Supplemental Figure S3: Ablations in *ex vivo* liver tissue evaluated immediately post H-FIRE treatment with the clinical MP and BP probes. The treatment protocol delivered 100x 3.5 kV bursts of the 2-1-2 waveform with each burst energized for 100 μs. (A) length and width, (B) ablation volume, and (C) circularity from ten (N=10) MP measurements and five (N=5) BP measurements. Values are reported as mean ± standard deviation.

the NanoKnife (AngioDynamics Inc., Latham, NY) ablation system: the "monopolar probe" (MP probe) and the "bipolar probe" (BP probe), respectively. The length of the exposed electrode was fixed to 1 cm to represent a common clinical electrode exposure for the MP probe and 0.7 cm to represent the fixed exposure for the BP probe. The center of the exposed electrode was 30 cm from the grounding pad in all simulations.

The effect of conductivity changes due to electroporation were modeled using a changing conductivity function using values determined experimentally by Neal et al.[11] with the initial and final conductivity set to 0.4113 and 0.927 S/m, respectfully. The transition between these two values was centered at the experimentally determined U87 reversible electroporation thresholds (Table 2) for the 50 μs monopolar (326 V/cm), 2 μs monopolar (400 V/cm), 2-1-0.25 (548 V/cm), 2-1-0.5 (587 V/cm), and 2-1-2 (865 V/cm) waveforms in separate simulations for each waveform. The electric field values along the x- and y-axis for each simulation were then exported into a spreadsheet. The experimentally determined lethal thresholds for U87 cells exposed to 50 μs monopolar (603 V/cm), 2 μs monopolar (688 V/cm), 2-1-0.25 (780 V/cm), 2-1-0.5 (925 V/cm), and 2-1-2 (1702 V/cm) waveforms were then used to find the x- and y-coordinates corresponding the expected ablations for each waveform. This data was then used to calculate the length, width, and volume for an ablation which would be theoretically created by each waveform.

Simulations of the MP probe were conducted using an applied voltage of 3000 V (Supplemental Table S1) to match the maximum output of the clinical NanoKnife IRE system and estimate the size of ablations possible if this system could deliver H-FIRE waveforms. Additional simulations were conducted using an applied voltage of 3500 V to approximate the experimental voltage measured from a prototype H-FIRE pulse generator with the MP probe (Supplemental Table S2) and the BP probe (Supplemental Table S3) in the validation experiments presented here.

Validation of Numerical Models

To validate these numerical models, predicted ablations were compared to ablations created in *ex vivo* liver tissue using the MP and BP NanoKnife electrodes individually in a single needle and grounding pad configuration. This configuration was tested by submerging freshly excised porcine liver in a water tank containing a 3g/L NaCl solution with a conductivity of 0.6 S/m. The tip of the probe was energized and a 0.254 mm aluminum sheet was placed 10 cm from the liver to serve as a current sink, simulating a distant grounding pad. All protocols used 100x 2-1-2 H-FIRE waveforms with each burst energized for 100 μs (Figure S2A) and all experiments were conducted within four hours post-mortem. An experimental high voltage pulse generator was used to deliver the H-FIRE treatments. The system was charged to its maximum voltage (4000 V) and the pulse output voltage and current were measured using 1000:1 and 10:1 voltage and current probes, respectively. A small amount of internal series resistance resulted in pulse amplitudes which were a few hundred volts below 4000 V and varied slightly as a function of the output current (Figure S2B).

Supplemental Table S1: Simulated ablation geometries created using experimentally determined lethal thresholds when 3.0 kV is delivered through a single 1 cm electrode (MP Probe) and a distal grounding pad. 3.0 kV is the maximum output of the clinical NanoKnife system.

50 µs Energized - 3.0 kV – MP Probe			
**Waveform**	**Width [cm]**	**Length [cm]**	**Volume [cm^3]**
50 µs mono	1.0	1.7	0.9
2 µs mono	0.9	1.6	0.7
2-1-0.25	0.8	1.6	0.5
2-1-0.5	0.7	1.5	0.4
2-1-2	0.4	1.3	0.1

100 µs Energized – 3.0 kV – MP Probe			
**Waveform**	**Width [cm]**	**Length [cm]**	**Volume [cm^3]**
100 µs mono	1.1	1.8	1.2
2 µs mono	1.1	1.7	1.0
2-1-0.25	1.0	1.7	1.0
2-1-0.5	0.9	1.6	0.6
2-1-2	0.5	1.4	0.2

The tissue was immediately sectioned and soaked in a 10 g/L tetrazolium chloride (TTC) solution for 20 minutes which dyed metabolically active regions red and exposed regions of tissue which had been irreversibly electroporated. The length (l) and width (w) of the ablation zones were then measured using calipers and values were reported as mean ± standard deviation. The circularity (c) of the ablations was calculated as $c = w / l$.

Ex Vivo Results versus Numerical Predictions

Experimental ablations were created in ex vivo porcine liver by delivering 100x 3.5 kV bursts of the 2-1-2 waveform (Supplemental Figure S2). Each burst was energized for 100 µs and bursts were delivered at a repetition frequency of 1 Hz. Experimental ablations created using the clinical MP probe with a 1 cm electrode exposure had an average length of 1.3 ± 0.3 cm and an average width of 0.7 ± 0.2 cm (Supplemental Figure S3). The numerical model of the MP probe predicted an ablation measuring 1.4 x 0.6 cm when these experimental conditions were simulated (Supplemental Table S2) and the results were within 0.1 cm of the average ablation created experimentally (1.3 x 0.7 cm). Experimental ablations created using the clinical BP probe had an average length of 1.4 ± 0.1 cm and an average width of 0.9 ± 0.2 cm (Supplemental Figure S3). The numerical model of the BP probe predicted an ablation measuring 1.2 x 0.7 cm using these experimental parameters (Supplemental Table S3) and the results were within 0.2 cm of the average ablation created experimentally (1.4 x 0.9 cm). This difference of one to two millimeters in length and width between the average experimental and predicted ablations was within serve as a preliminary validation of the ability of these models, which incorporate dynamic tissue conductivity with reversible and lethal thresholds found in vitro, to predict ablation dimensions in tissue.

Supplemental Table S2: Simulated ablation geometries created using experimentally determined lethal thresholds when 3.5 kV is delivered through a single 1 cm electrode (MP Probe) and a distal grounding pad. 3.5 kV was the average output voltage measured using an experimental H-FIRE system.

50 µs Energized – 3.5 kV – MP Probe

Waveform	Width [cm]	Length [cm]	Volume [cm^3]
50 µs mono	1.1	1.8	1.2
2 µs mono	1.0	1.7	0.9
2-1-0.25	0.9	1.6	0.7
2-1-0.5	0.8	1.5	0.5
2-1-2	0.5	1.3	0.2

100 µs Energized – 3.5 kV – MP Probe

Waveform	Width [cm]	Length [cm]	Volume [cm^3]
100 µs mono	1.3	1.9	1.6
2 µs mono	1.2	1.8	1.4
2-1-0.25	1.2	1.8	1.3
2-1-0.5	1.0	1.7	0.8
2-1-2	0.6	1.4	0.3

Supplemental Table S3: Simulated ablation geometries created using experimentally determined lethal thresholds when 3.5 kV is delivered through a single 0.7 cm electrode (BP Probe) and a distal grounding pad. 3.5 kV was the average output voltage measured using an experimental H-FIRE system.

50 µs Energized – 3.5 kV – BP Probe

Waveform	Width [cm]	Length [cm]	Volume [cm^3]
50 µs mono	1.2	1.5	1.1
2 µs mono	1.1	1.5	0.9
2-1-0.25	1.0	1.4	0.7
2-1-0.5	0.9	1.3	0.5
2-1-2	0.7	1.1	0.2

100 µs Energized – 3.5 kV – BP Probe

Waveform	Width [cm]	Length [cm]	Volume [cm^3]
100 µs mono	1.3	1.7	1.5
2 µs mono	1.3	1.6	1.4
2-1-0.25	1.2	1.6	1.3
2-1-0.5	1.1	1.5	0.9
2-1-2	0.7	1.2	0.3

Supplemental References

1. Miklavcic, D., Semrov, D., Mekid, H. & Mir, L.M. A validated model of in vivo electric field distribution in tissues for electrochemotherapy and for DNA electrotransfer for gene therapy. *Biochim Biophys Acta* **1523**, 73-83 (2000).
2. Davalos, R.V., Mir, L.M. & Rubinsky, B. Tissue ablation with irreversible electroporation. *Annals of Biomedical Engineering* **33**, 223-231 (2005).
3. Sel, D., *et al.* Sequential finite element model of tissue electropermeabilization. *IEEE Transactions on Biomedical Engineering* **52**, 816-827 (2005).

4. Neal, R.E., *et al.* In Vivo Irreversible Electroporation Kidney Ablation: Experimentally Correlated Numerical Models. *Biomedical Engineering, IEEE Transactions on* **62**, 561-569 (2015).
5. Garcia, P.A., *et al.* Intracranial nonthermal irreversible electroporation: in vivo analysis. *J Membr Biol* **236**, 127-136 (2010).
6. Rossmeisl Jr, J.H., *et al.* Safety and feasibility of the NanoKnife system for irreversible electroporation ablative treatment of canine spontaneous intracranial gliomas. *Journal of neurosurgery* **123**, 1008-1025 (2015).
7. Sel, D., Lebar, A.M. & Miklavcic, D. Feasibility of employing model-based optimization of pulse amplitude and electrode distance for effective tumor electropermeabilization. *IEEE transactions on biomedical engineering* **54**, 773-781 (2007).
8. Zupanic, A., Kos, B. & Miklavcic, D. Treatment planning of electroporation-based medical interventions: electrochemotherapy, gene electrotransfer and irreversible electroporation. *Physics in medicine and biology* **57**, 5425 (2012).
9. Miklavcic, D., *et al.* Towards treatment planning and treatment of deep-seated solid tumors by electrochemotherapy. *BioMedical Engineering OnLine* **9**, 1 (2010).
10. Sharabi, S., *et al.* Dynamic effects of point source electroporation on the rat brain tissue. *Bioelectrochemistry* **99**, 30-39 (2014).
11. Neal, R.E., *et al.* In vivo characterization and numerical simulation of prostate properties for non-thermal irreversible electroporation ablation. *The Prostate* **74**, 458-468 (2014).
12. Martin, R.C., Schwartz, E., Adams, J., Farah, I. & Derhake, B.M. Intra - operative Anesthesia Management in Patients Undergoing Surgical Irreversible Electroporation of the Pancreas, Liver, Kidney, and Retroperitoneal Tumors. *Anesth Pain Med* **5**, e22786 (2015).
13. McGrath, C.D. & Hunter, J.M. Monitoring of neuromuscular block. *Contin Educ Anaesth Crit Care Pain* **6**, 7-12 (2006).
14. Thomson, K.R., *et al.* Investigation of the safety of irreversible electroporation in humans. *J Vasc Interv Radiol* **22**, 611-621 (2011).
15. Rogers, W.R., *et al.* Strength-duration curve for an electrically excitable tissue extended down to near 1 nanosecond. *IEEE transactions on plasma science* **32**, 1587-1599 (2004).
16. Reilly, J.P., Freeman, V.T. & Larkin, W.D. Sensory effects of transient electrical stimulation--evaluation with a neuroelectric model. *IEEE Trans Biomed Eng* **32**, 1001-1011 (1985).
17. Rogers, W.R., *et al.* Strength-duration curve for an electrically excitable tissue extended down to near 1 nanosecond. *Plasma Science, IEEE Transactions on* **32**, 1587-1599 (2004).
18. Arena, C.B., *et al.* High-frequency irreversible electroporation (H-FIRE) for non-thermal ablation without muscle contraction. *Biomed Eng Online* **10**, 102 (2011).
19. Sano, M.B., *et al.* Bursts of Bipolar Microsecond Pulses Inhibit Tumor Growth. *Scientific reports* **5**(2015).
